# Distribution, Characteristics, and Regulatory Potential of Long Noncoding RNAs in Brown-Rot Fungi

**DOI:** 10.1155/2019/9702342

**Published:** 2019-05-02

**Authors:** Alessandra Borgognone, Walter Sanseverino, Riccardo Aiese Cigliano, Raúl Castanera

**Affiliations:** ^1^Sequentia Biotech, Edifici Eureka, Campus UAB, Cerdanyola del Vallès, Barcelona, Spain; ^2^Center for Research in Agricultural Genomics, CRAG (CSIC-IRTA-UAB-UB), Campus UAB, Cerdanyola del Vallès, Barcelona, Spain

## Abstract

Long noncoding RNAs have been thoroughly studied in plants, animals, and yeasts, where they play important roles as regulators of transcription. Nevertheless, almost nothing is known about their presence and characteristics in filamentous fungi, especially in basidiomycetes. In the present study, we have carried out an exhaustive annotation and characterization of lncRNAs in two lignin degrader basidiomycetes, *Coniophora puteana* and *Serpula lacrymans.* We identified 2,712 putative lncRNAs in the former and 2,242 in the latter, mainly originating from intergenic locations of transposon-sparse genomic regions. The lncRNA length, GC content, expression levels, and stability of the secondary structure differ from coding transcripts but are similar in these two species and resemble that of other eukaryotes. Nevertheless, they lack sequence conservation. Also, we found that lncRNAs are transcriptionally regulated in the same proportion as genes when the fungus actively decomposes soil organic matter. Finally, up to 7% of the upstream gene regions of *Coniophora puteana* and *Serpula lacrymans* are transcribed and produce lncRNAs. The study of expression trends in these gene-lncRNA pairs uncovered groups with similar and opposite transcriptional profiles which may be the result of *cis*-transcriptional regulation.

## 1. Introduction

In recent years, RNA sequencing (RNA-seq) has emerged as a powerful technology for genome-wide expression profiling. Advancements in high-throughput sequencing approaches and reduction in costs led to the discovery of large amounts of RNA transcripts, including novel classes of non-protein-coding transcripts. This revolutionary breakthrough helped to unmask the noncoding fraction of eukaryotic transcriptomes, allowing the identification of long noncoding RNAs (lncRNAs) [1], a new functional class of RNAs pervasively transcribed in a wide range of genomes—from yeasts to animals. lncRNAs are generally defined as 200 nt or longer transcripts, mostly transcribed by RNA polymerase II and characterized by very low or no coding potential [2, 3]. Similarly to the canonical structural processing of mRNA molecules, some lncRNAs are characterized by 5′ capping, alternative splicing, polyadenylation, and posttranscriptional modifications [4], but usually have little or no open reading frames (ORF) [5]. They can be transcribed from either strand and are often classified based on their genome location and context as sense, antisense, bidirectional, intronic, or intergenic [6, 7]. An initial debate referred to these functional molecules as transcriptional “noise” [8]. However, in the last decades lncRNAs have gained importance as a heterogeneous group of noncoding transcripts involved in diverse regulatory processes. As an example, recent experimental findings indicated that *Xist* lncRNA is strictly required for X chromosome inactivation in animals [9], highlighting the key role of lncRNAs in developmental processes such as embryogenesis and cell differentiation [10]. In addition, their essential involvement in diverse cellular mechanisms associated to transcriptional and posttranscriptional regulation, protein translation, and chromatin modification [11, 12], among others, is becoming evident. While many research efforts have been made to decipher the role of lncRNAs, less is known about their origin and precise molecular function. A number of hypotheses attempted to elucidate the evolutionary pattern that led to the emergence of lncRNAs, suggesting their possible origin from alterations of protein-coding genes, modification of transposable elements, and activation of untranscribed DNA sequences [13, 14]. The faster evolutionary turnover of lncRNAs compared to coding gene sequences and low degree of interspecies conservation across the eukaryotic domain [15] made difficult the identification of relationships between lncRNAs in organisms that do not share a common evolutionary origin. Up to now, most conservation studies were based on the analysis of primary sequence. Nevertheless, more comprehensive approaches attempted to integrate information from secondary structures and the functional role of lncRNAs to characterize the evolutionary conservation on a global scale [15]. These strategies are particularly relevant in light of the tight correlation between structure and function of most lncRNAs. This emerged, for instance, by the discovery of their physical associations (in *cis* or *trans*) with chromatin complexes required for the epigenetic regulation of target regions [7, 16]. Considering the increasing number of lncRNAs characterized across eukaryotic genomes [17–19], important efforts have been made by researchers to describe and validate their functional role. While the discovery and characterization of lncRNAs in the animal and plant kingdoms is a current topic [18, 19], their abundance, features, and functional role remain poorly investigated in fungi. Attempts to categorize lncRNAs in fungal species have been based on different criteria, such as mode of action in *trans* or *cis*, functional context, or additional properties [17]. In the last years, studies carried out in the budding yeast *Saccharomyces cerevisiae* and fission yeast *Schizosaccharomyces pombe* have elucidated the crucial regulatory role of lncRNAs in complex biological mechanisms, which can act by interfering with the expression of sense or antisense proximal located genes. In budding yeasts, lncRNAs involved in the regulation of sexual sporulation can act by blocking aberrations during germ cell differentiation and interfering with sense transcription of genes involved in meiotic division [20, 21]. Although no evidence of such conserved regulatory mechanisms has been found in fission yeast, lncRNAs also play crucial roles during the meiotic development in these species, by blocking RNA elimination factors that repress meiotic gene transcription [22, 23], facilitating proper homologous chromosome pairing [24], and mediating the progression instead of the induction of meiosis [25]. Beside meiosis and mitosis, other regulatory strategies of lncRNA are associated to a number of cellular processes in yeasts, such as cell-cell adhesion during filament formation of cells in response to nutrient starvation [26], regulation of phosphate metabolism in [27], stress response [28], and telomere synthesis and maintenance [29, 30]. Fungal lncRNAs were also screened in other ascomycetes such as *Neurospora crassa* [31] and *Fusarium graminearum* [32], but the first functional characterization of a lncRNA in filamentous fungi (*HAX1*) was achieved only very recently in the saprophyte *Trichoderma reesei* [33]. Contrarily to the repressive effects on transcription described for most lncRNAs, *HAX1* acts as an activator in the regulatory network of *T. reesei* cellulase expression. Also, the tight link observed between a strain-specific variation of *HAX1* length and its regulatory activity suggested a unique strategy for *HAX1* dynamics [33]. To date, several studies have explored the presence of lncRNAs in ascomycetes, but very little is known about the distribution and properties of lncRNAs in basidiomycetes. A genome-wide characterization in the white-rot *Ganoderma lucidum* identified a total of 402 putative lincRNAs (long intergenic noncoding RNAs), using RNA-seq data obtained from three developmental stages combined with a modified 3′ RACE method to determine the transcriptional direction of a transcript. These findings revealed the coexpression of a lincRNA subset with adjacent protein-coding genes, suggesting a potential lincRNA-mediated transcriptional regulation of genes involved in wood degradation, triterpenoid biosynthesis, and mating in *G. lucidum* [34].

In this study, we carry out an in silico genome-wide characterization of lncRNAs in the basidiomycete fungi *Coniophora puteana* and *Serpula lacrymans* (also known as “cellar” and “dry-rot” fungi, respectively) using transcriptomic data obtained from previous RNA-seq experiments. In their natural habitat, *C. puteana* and *S. lacrymans* produce brown-rot decay on the wood of conifers and occasionally on hardwood trees. Additionally, both fungi are widely known because they cause damage on buildings and construction materials. In the last years, the genomes of a diverse group of Boletales species have been sequenced and annotated and shown to display important differences in terms of genome size and gene content [35, 36]. Moreover, a recent study focused on the transposon landscape in Boletales showed that TEs, especially LTR retrotransposons, have undergone differential expansions leading to a great genomic variability in this basidiomycete order [37].

## 2. Materials and Methods

### 2.1. Genome and Transcriptome Data Sources

*S. lacrymans* and *C. puteana* raw mRNA-seq reads were obtained from SRA database BioProject accession PRJNA272430. The data corresponds to mRNA obtained from mycelia cultured for 7 days at 18°C in the dark in two different media using 3 biological replicates: (i) MMN (Melin–Norkrans minimal medium) and (ii) SOM (soil organic matter) extract (see [38] for details). This last medium consists of an MMN medium without nitrogen induced with a SOM extract obtained from upper soil layer.

### 2.2. lncRNA Detection and Classification

**Figure 1:**
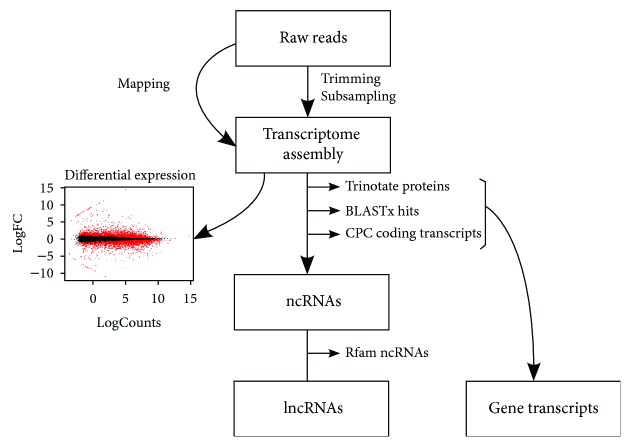
Pipeline of lncRNA annotation.

FastQC was used for evaluating read quality and BBDuk tool from the BBMap package (https://sourceforge.net/projects/bbmap/) for adapter and quality trimming. A total of 77 clean million reads (50 bp) obtained by merging replicates of the two conditions were pooled and assembled with Trinity [39] (200 bp length cut-off), in order to obtain the reference transcriptomes of *S. lacrymans* and *C. puteana*. The pipeline shown in Figure 1 was used for identifying lncRNAs. Briefly, TransDecoder [39] was run to identify candidate coding regions, which were filtered out. BLASTX searches [40] vs. each of the species reference annotation available in the Mycocosm database [41] were run with the remaining transcripts to identify known coding regions not detected by TransDecoder (i.e., small gene fragments). The remaining transcripts were scanned with CPC (coding potential calculator) [42], retaining only those classified as noncoding. This dataset was then used as query against Rfam database using Infernal software [43] to remove known noncoding RNAs other than lncRNAs. The remaining noncoding transcripts were mapped to the genome with gmap [44] with a 90% identity cut-off, and only the best hit was reported.

### 2.3. Characterization of Minimum Free Energy

Minimum free energy (MFE) values for lncRNAs and protein-coding RNAs were estimated using the RNAfold algorithm from the ViennaRNA package [45]. The program was run using the following parameters: -p –d2 –noLP. MFE of 1,000 randomly selected lncRNAs and protein-coding transcripts were compared after normalizing by transcript length.

### 2.4. Transcriptome Quantification

Libraries were mapped back to the corresponding transcriptome assembly using Bowtie2 [46], and expression levels of annotated features were calculated using RSEM [47] in TMM-normalized TPM values (transcripts per million). Differential expression of lncRNAs between the two conditions was obtained with EdgeR [48] using a false discovery rate < 0.05 and a LogFC > 1.

### 2.5. Interspecies Conservation of lncRNA

All-by-all BLASTN followed by MCL (Markov cluster) clustering [49] (inflation value = 2) was performed with *S. lacrymans* and *C. puteana* putative lncRNAs. Conserved lncRNAs were identified by looking for clusters with members of both species.

## 3. Results and Discussion

### 3.1. Genome-Wide Identification of lncRNAs in *C. puteana* and *S. lacrymans*

We assembled a total of 19,604 and 14,103 isoforms in *C. puteana* and *S. lacrymans,* which were later mapped to their respective genomes. We observed that the assembled transcripts overlapped with 80.1 and 82% of the genes present in the reference annotations of *C. puteana* and *S. lacrymans* [35, 50]. In addition, 77.8% and 86.9% of these overlapping transcripts were potentially full-length (according to a reciprocal overlap between reference gene and assembled isoform higher than 80% of their respective lengths). Considering that some annotated genes have no transcript support (i.e., gene models built based only on homology or structural evidence) and some others might not be expressed in the tested conditions, this evidence suggests that our assemblies captured a very important fraction of the transcriptomes. Nevertheless, the assembly of *S. lacrymans* was slightly more comprehensive than that of *C. puteana*. In addition, we found that only 5.3 and 4.5% of the assembled coding transcripts (Figure 1, “gene transcripts”) did not overlap with genes in *C. puteana* and *S. lacrymans*, respectively. These transcripts may represent gene fragments or actual genes specifically expressed in the conditions assayed. Also, the low number of transcripts in this category evidences that the official annotations are very exhaustive. After discarding coding transcripts as well as other types of noncoding RNAs using our pipeline, we identified 2,712 lncRNAs in *C. puteana* and 2,242 in *S. lacrymans*, (Supplementary Datasets S1 and S2). The vast majority of lncRNAs originated from intergenic regions in both species (76 and 85% in *C. puteana* and *S. lacrymans*, respectively), whereas 14 and 22% overlapped with exons, and less than 2% were intronic (Table 1). A previous genome-wide study focused on lncRNAs in the basidiomycete *G. lucidum* reported 402 long intergenic ncRNAs [34]. Also, a study carried out in *Cryptococcus neoformans* identified 1,197 transcribed regions without coding potential ranging from 106 to 5,555 nt [51]. In ascomycetes, recent studies identified a similar content of lncRNA candidates, as described for *F. graminearum* (547 developmental stage-specific lncRNAs) [32] and *N. crassa* (939 and 1478 lincRNAs [31, 52]). In *S. cerevisiae*, approximately 2,000 lncRNAs have been identified thus far and their presence and abundance have been linked to different growing conditions [53, 54]. In this sense, it should be noted that the gene content and genome size of the latter species are much lower than *C. puteana* or *S. lacrymans*, and thus the density of lncRNAs is clearly higher in spite of the similar number of lncRNAs detected. Our results are in line with the very few findings dealing with basidiomycetes lncRNAs and suggest that these transcriptional units represent a much smaller fraction of the fungal transcriptome than the coding transcripts. This contrasts to what has been described for humans, where up to 68% of the genes are noncoding [55] and might be explained by the shorter intergenic distances of fungal genomes in comparison to other eukaryotes, which are the main source of lncRNAs.

### 3.2. *C. puteana* and *S. lacrymans* Elements Share the Signatures of Canonical Eukaryotic lncRNAs

**Figure 2:**
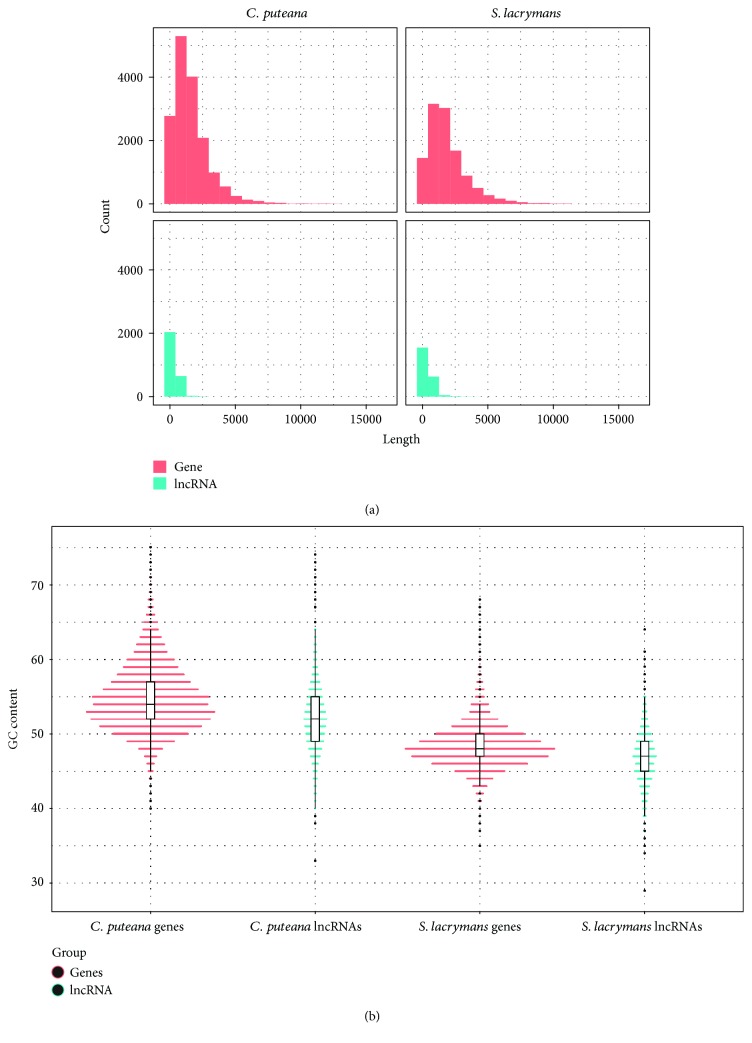
Distribution of length (a) and percentage of GC content (b) of coding isoforms and lncRNAs in *C. puteana* and *S. lacrymans*.

**Figure 3:**
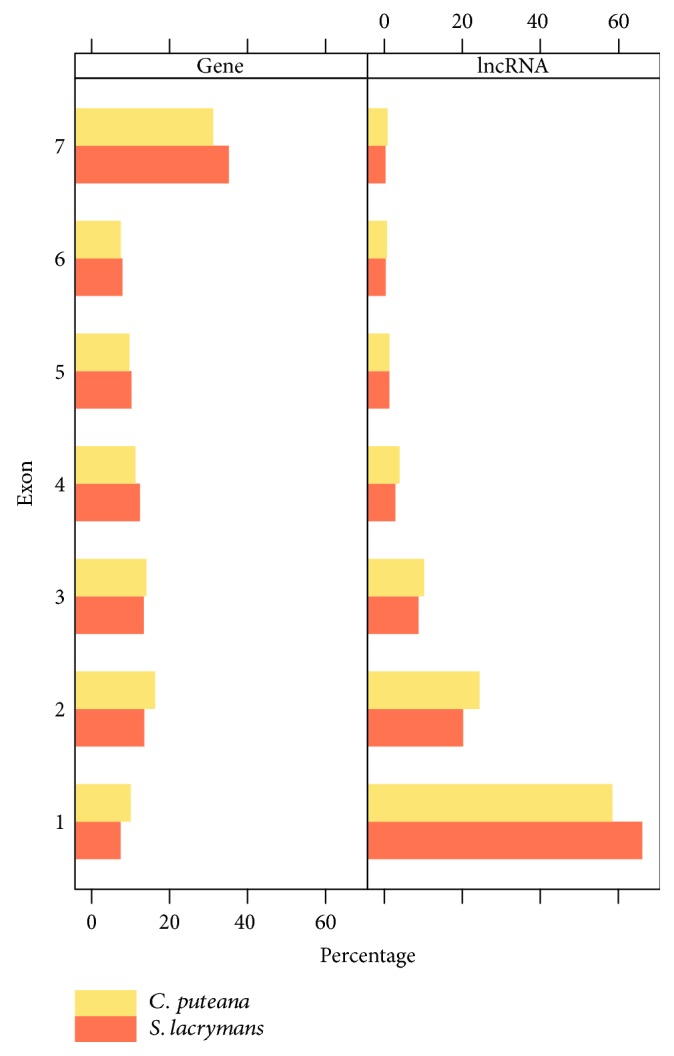
Exon number of lncRNAs and coding isoforms in *C. puteana* (orange) and *S. lacrymans* (yellow).

The vast majority of lncRNAs were shorter than 500 nt in both species (Figure 2(a)), with *S. lacrymans* lncRNAs being longer than those of *C. puteana* (mean of 434 nt vs. 377 nt, Wilcoxon *p* value = 1.288*e*-08). The same pattern was found on coding sequences (1,889 nt vs. 1,612 nt), although we cannot rule out the possibility that these small but significant differences are due to the assembly process. Eukaryotic lncRNAs are far shorter than genes and by definition are longer than 200 nt, although noncoding transcripts much longer that this threshold are sometimes found. In example, a study analyzing lncRNAs in the basidiomycete *Ganoderma lucidum* reported an average length of 609 nt and an important fraction of lncRNAs longer than 1,000 bp [34]. In terms of GC content, lncRNAs of both species showed a lower content than genes (Figure 2(b)). This phenomenon has been linked to a higher accessibility for putative interactions with cellular factors [56]. *S. lacrymans* lncRNAs displayed the lowest CG content, with an average GC value per element of 48.5% vs. the 54.9% found for *C. puteana*. This finding does not only apply to lncRNAs, as coding sequences follow the same trend. In addition, lncRNAs from both species were mainly monoexonic (60-65%) whereas 20-25% displayed 2 exons (Figure 3). In fact, as exon length was similar in lncRNAs and genes of both species (ranging from 239 to 275 nt in *C. puteana* and 250 to 261 in *S. lacrymans*), the differences in transcript length between the lncRNAs and genes could be mainly due to the higher exon number of coding elements. Similar features were described in the ascomycete *F. graminearum* during perithecial development, which displayed a much higher number of monoexonic lncRNAs than mRNAs (~70% vs. 20%) and transcript lengths ranging from 200 to 2,000 nt approximately. In addition, the AU content of lncRNAs in this filamentous fungus was higher than coding sequences of mRNAs, but slightly lower than intergenic regions [32].

**Figure 4:**
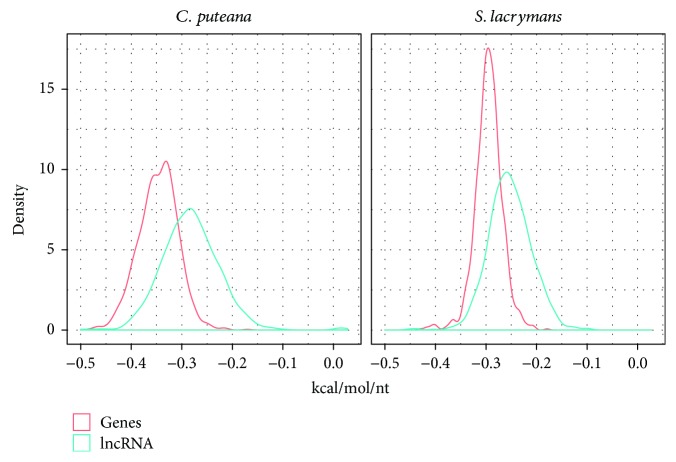
Minimum free energy (kcal/mol/nt) of 1,000 random lncRNAs and coding transcripts normalized by length.

Previous studies in humans and plants have shown that lncRNAs are substantially less folded than mRNAs [57, 58]. The stability of a secondary structure and backbone conformation of an RNA molecule is determined by its minimum free energy (MFE), with lower energy values being associated to more stable structures [59]. To estimate the MFE of putative lncRNAs and compare it with that of coding transcripts, we applied the RNAfold algorithm [45] to 1,000 randomly selected transcripts from each group (lncRNAs and coding), and the results were corrected by the sequence length. We found that lncRNAs display higher corrected MFE than do genes in both species (Figure 4, Wilcoxon *p* value = 2.2*e*-16), indicative of a lower thermodynamic stability. As reported in previous studies, a secondary structure of lncRNAs tends to have less stable conformation and consequently higher free energy than do protein-coding transcripts [57]. Similar findings were observed in the plant pathogen oomycete *Phytophthora sojae*, where 940 identified lncRNAs displayed higher MFE mean values when compared to protein-coding genes (-191 vs. -214 kcal/mol) [60]. In spite of the similar characteristics found in *S. lacrymans* and *C. puteana* lncRNAs in terms of quantity, length, exon number, or secondary structure stability, we found almost no sequence conservation among them. After clustering by similarity all the sequences using all-by-all BLASTN followed by MCL, we found only three small clusters displaying lncRNAs from the two species (one with three sequences and the remaining with only two). The low sequence conservation of lncRNAs has been long known in plants and animals, although this phenomenon has not been investigated in fungi due to the limited amount of available information. A hypothesis to explain such low conservation levels is that the selection may act on structure rather than on primary sequence [61]. Nevertheless, further experimental work will be needed to demonstrate whether this phenomenon occurs in filamentous fungi. It has been proposed that the low similarity of lncRNAs identified in *F. graminearum* with those found in other eukaryotes might reflect the nonexhaustive annotation status of lncRNAs in filamentous fungi or the high sequence divergence in fungal lncRNA [32]. In this sense, our results point to a general lack of sequence conservation of lncRNAs in the fungal kingdom. The estimated divergence between the two species was dated at 84 million years ago [36], but as brown-rot fungal species they share a lifestyle and have a conserved lignin-degrading machinery based on common extracellular enzymes [62, 63]. Nevertheless, the important differences found in the dynamics of transposable elements [37] and the absence of lncRNA conservation suggest that the evolution of the noncoding genome is highly divergent in these two species.

### 3.3. Transposon-Associated lncRNAs

The coordinates of annotated lncRNAs were intersected with REPET TE annotations of both species [37] to uncover the presence of TE-associated lncRNAs. In the case of *C. puteana*, only 62 out of the 2,712 annotated lncRNAs overlapped with an annotated TE. A total of 40 TE families had at least one lncRNA overlapping with any of their members. Remarkably, 75% of these TE families producing lncRNAs were DNA elements, especially TIR (terminal inverted repeat) elements and MITEs (miniature inverted-repeat transposable elements). In *S. lacrymans*, we found 64 out of the 2,242 lncRNAs overlapping TEs (38 TE families in total), with the same prevalence of DNA transposons over RNA transposons (63% of the TE families of the DNA class). This amount of TE-associated lncRNAs is much lower than that reported for plants or animals, where 20-80% of the lncRNAs have a TE origin [13, 64, 65]. This fact is even more striking in the case of *S. lacrymans*, as this species has undergone a dramatic amplification of RNA transposons during the last 5 million years, especially of LTR retrotransposons in the Gypsy superfamily [37]. LTR retrotransposons are the most prevalent TE order in fungi [66], and also in most plants [67]. Their ability to efficiently colonize host genomes reside in their copy-and-paste mechanism (in contrast to the cut-and-paste mechanism of most DNA transposons) but also in the fact that they tend to accumulate in gene-poor heterochromatic regions. Most of the LTR retrotransposons of *S. lacrymans* are clustered in transcriptionally silent regions displaying very few lncRNAs (Supplementary Figure S3). Previous studies in other basidiomycetes such as *Coprinopsis cinerea*, *Laccaria bicolor*, or *Pleurotus ostreatus* have demonstrated that transposon-rich regions are specifically targeted by DNA methylation and siRNAs, which efficiently shut down their expression [68, 69]. In this sense, the lack of lncRNAs in the repeat-rich regions of the genome seems to be a side effect of the arms race between TEs and fungal genome defense mechanisms.

### 3.4. Transcriptional Profiles of lncRNA

**Figure 5:**
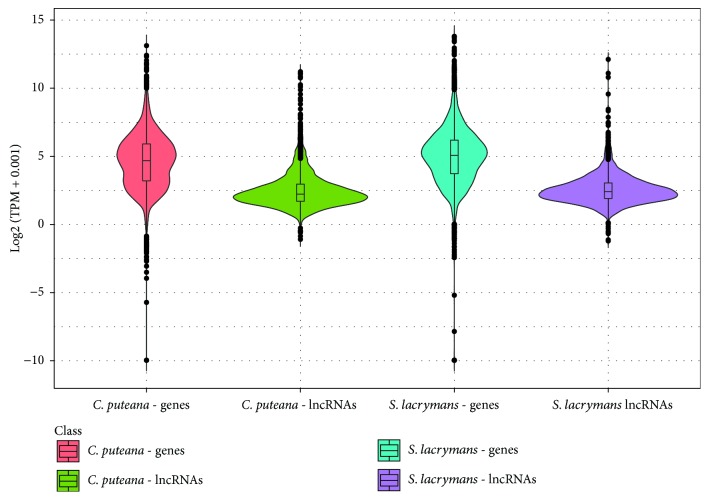
Distribution of transcriptional levels of coding transcripts and lncRNAs of *C. puteana* and *S. lacrymans* cultured on SOM medium. Box plots inside the violin plots are presented, showing the median value as a horizontal line.

The expression levels of lncRNAs were lower than those of coding transcripts (Figure 5). As an example, in SOM medium, *S. lacrymans* lncRNAs had an average expression of 12.8 TPM vs. 89.2 TPM of coding transcripts. In the same conditions, *C. puteana* lncRNAs displayed an average of 15.6 TPM vs. 58.9 TPM of coding transcripts. This phenomenon has been previously described for many plant and animal lncRNAs, and it is in fact considered a canonical feature of these pervasive transcripts [2]. The global distribution of transcription levels was very similar in *C. puteana* and *S. lacrymans* and independent of the culture media used (Figure 5, Supplementary Figure S4). In addition, the expression of lncRNAs was found to be more uniform than that of genes, with the levels of most transcripts concentrated around the median of the population (Figure 5). In addition to the general expression levels, we performed differential expression analyses to identify lncRNAs showing a significant increase or decrease in expression when the fungus grows on media with organic matter extracts (SOM medium). Using a log2-fold change cutoff of 1 and an FDR adjusted *p* value <0.05, we identified 341 (12.6%) differentially expressed lncRNAs between the two conditions in *C. puteana* and 210 (9.4%) in *S. lacrymans*. A total of 184 lncRNAs were upregulated in SOM medium and 157 downregulated in the former, whereas the latter showed 89 SOM-upregulated lncRNAs and 121 downregulated. The proportions of DE-lncRNAs are similar or even higher than those found for coding transcripts using the same cutoff (9.9% in *C. puteana* and 10.0% in *S. lacrymans*). The presence of differential transcription suggests that these elements are likely functional and not simply the result of transcriptional noise. Differentially expressed lncRNAs have been widely studied in humans, where a fraction of them show transcriptional profiles associated to different cancer types [55]. In plants, many lncRNAs show stage-specific transcriptional profiles, suggesting a putative role during reproductive development [70]. In fungi, a study reported the presence of 11 lncRNAs upregulated in response to light, whereas no differential expression was found in response to temperature [52]. It is well-known that fungi adjust their transcriptome in response to nutrient stimuli. In example, *S. cerevisiae* undergoes massive transcriptional reprogramming as a consequence of activation and repression of genes involved in the metabolism of sugars other than glucose [71]. Also, Shah et al. report that 16 to 29% of the genes of a set of 10 brown-rot and ectomycorrhizal fungi were upregulated in SOM media [38]. According to our results, the noncoding transcriptome is also regulated in response to nutrient availability. We speculate that the presence of differentially expressed lncRNAs in *C. puteana* and *S. lacrymans* in SOM media may underlie a function of these elements when the fungus is under active decomposition of soil organic matter.

### 3.5. lncRNAs in Brown-Rot Fungi: A Source of *cis*-Regulatory Elements?

**Figure 6:**
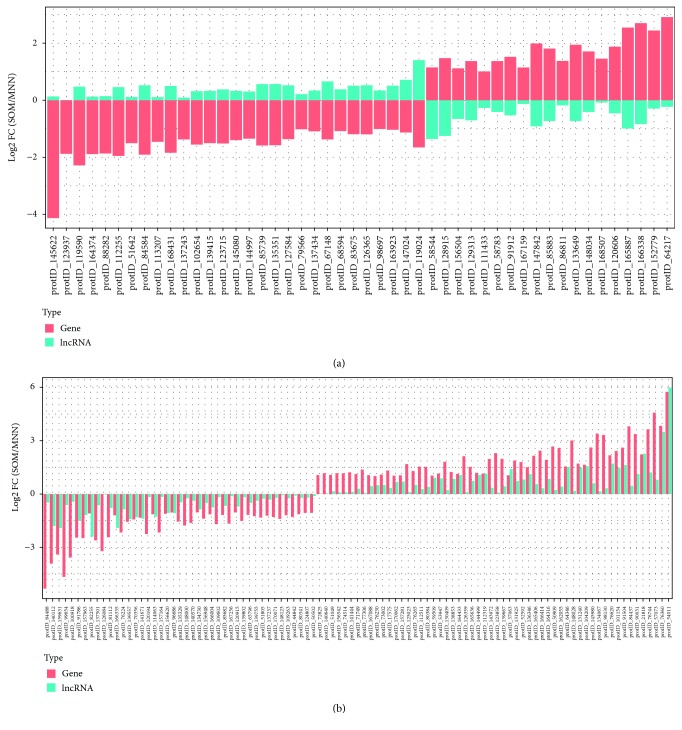
Expression profiles of 147 *C. puteana* DE genes and their linked upstream lncRNAs, showing opposite (a) and same (b) expression trends in SOM vs. minimal MNN media.

In order to investigate the *cis*-regulatory potential of our annotated lncRNAs, we analyzed their position in respect of the reference annotations of *C. puteana* [35] and *S. lacrymans* [50]. We found that up to 1,011 *C. puteana* genes and 902 S*. lacrymans* genes of their corresponding reference annotations have a lncRNA at less than 500 bp upstream the transcription start site (hereafter referred as “lncRNA associated genes,” Supplementary Table S6). To test if these genes could be under the regulation of the upstream lncRNA, we used the differentially expressed genes between SOM and MNN media published by Shah et al. [38] (original transcriptome datasets analyzed with *C. puteana* and *S. lacrymans* reference annotations) in both species and compared the gene expression trends (log2FC) with those of our lncRNAs. The aim was to analyze if the transcriptional response of the genes to the culture media was the same or the contrary than its upstream lncRNA. In *C. puteana*, we found that 147 out of the 1,011 lncRNA-associated genes were differentially expressed (log2FC cutoff = 1, FDR adjusted *p* value <0.05). The analysis of the corresponding 147 gene-lncRNA pairs revealed 48 of them having an opposite expression and 99 of them showing the same expression profile (Figure 6). In the case of *S. lacrymans*, 120 out of the 902 lncRNA-associated genes were differentially expressed. After a closer analysis of the 120 gene-lncRNA pairs, we found 45 showing an opposite expression and 73 with the same expression trend (Supplementary Figure S5). lncRNAs are known *cis*-regulatory elements in plants and animals. The most recent literature suggests that the expression of lncRNA is usually highly correlated (either positively or negatively) with that of their surrounding genes. Positive correlations might result from common regulations of local chromatin or actual lncRNA regulation [70]. By contrast, the overexpression of a lncRNA may also lead to a downregulation in the expression of adjacent genes by physical interference with transcription or by overlap with the proximal gene [17]. In yeasts, the expression of the *HO* gene has been experimentally demonstrated to be regulated by the expression of a lncRNA in the promoter region by displacement of a cell-cycle box binding factor [72]. In basidiomycetes, the first functional characterization of a lncRNA was recently published in the human pathogen *Cryptococcus*, where *RZE1* was demonstrated to control yeast-to-hypha transition, through the transcriptional *cis*-regulation in of the downstream gene *ZNF2* [73]. Our results indicate that the upstream regions of about 7% of *C. puteana* and *S. lacrymans* genes are transcribed in the form of lncRNAs, and some of these genes might be positively or negatively regulated by them. In a genome-wide study carried out in the white rot basidiomycete *G. lucidum*, the authors also found positive and negative correlations between lncRNAs and surrounding genes, some of them coding for enzymes involved in wood degradation [34]. When we analyzed the function of DE genes under potential lncRNA regulation, we found that their associated GO terms were related to proteolysis and carbohydrate metabolism, which are typically enriched in the secretome of lignin-degrading species [37]. Nevertheless, the enrichment of most of these GO terms was not significant after correction for multiple testing (Table 2). In agreement with this finding, oxidoreductases, hydrolases, and peptidases were described as the most upregulated secreted enzyme genes during brown-rot and ectomycorrhizal fungi growth on SOM media [38]. In this sense, future experimental work will be needed to understand the link between transcription of basidiomycete lncRNAs and their linked genes, especially those involved in wood degradation.

## 4. Conclusions

In the present study, we have uncovered the presence of 2,712 and 2,242 putative lncRNAs in the basidiomycetes *C. puteana* and *S. lacrymans*, mainly arising from intergenic regions. The length, GC content, expression levels, and minimum free energy of these transcripts are conserved between the two species and resemble that of other eukaryotes. Nevertheless, they lack sequence conservation. TE-associated lncRNAs were found to be present in a much lower proportion than in plant and animals, and those detected predominantly derive from DNA elements. Finally, we found that up to 7% of the genes of*C. puteana*and*S. lacrymaNS* carry a lncRNA in the upstream region, and a fraction of them were differentially expressed in the two conditions tested. The analysis of expression trends in these gene-lncRNA pairs uncovered groups with similar and opposite transcriptional profiles which may be the result of *cis*-transcriptional regulation. In summary, our study provides a detailed annotation and in silico characterization of putative lncRNAs in two well-studied brown-rot basidiomycetes. This information can be used as a starting point for the experimental validation and functional description of these enigmatic transcriptional units in basidiomycete fungi.

## Figures and Tables

**Table 1:** Classification of assembled transcripts in *C. puteana* and *S. lacrymans.*

Feature	*C. puteana*	*S. lacrymans*
Isoform	19,604	14,103
Isoform clusters^∗^	17,208	12,543
Coding isoform	16,279	11,345
lncRNA (all)	2,712	2,242
lncRNA intergenic	2,059	1,912
lncRNA overlapping exon	607	314
lncRNA intronic	46	16

^∗^Cluster of isoforms originating from a single locus.

**Table 2:** Top ten enriched terms in *C. puteana* and *S. lacrymans* DEG genes with lncRNAs in their promoter region.

Species	GO	Category	GO name	Ratio in DEG genes	Ratio in genome	*p* value	*p* value fdr
*C puteana*	GO:0006508	BP	Proteolysis	8,8	2,1	1, 19*E* − 05	0,006
	GO:0005975	BP	Carbohydrate metabolic process	6,1	1,4	0,000202	0,076
	GO:0006810	BP	Transport	8,8	2,8	0,000224	0,076
	GO:0000272	BP	Polysaccharide catabolic process	1,4	0,0	0,00165	0,3
	GO:0004190	MF	Aspartic-type endopeptidase activity	4,8	0,7	5, 65*E* − 05	0,018
	GO:0004553	MF	Hydrolase activity, hydrolyzing O-glycosyl compounds	4,8	0,9	0,000315	0,09
	GO:0005215	MF	Transporter activity	6,1	1,7	0,000865	0,202
	GO:0008810	MF	Cellulase activity	1,4	0,0	0,00111	0,228
	GO:0004338	MF	Glucan exo-1,3-beta-glucosidase activity	1,4	0,1	0,00304	0,414
	GO:0030246	MF	Carbohydrate binding	2,0	0,2	0,00472	0,54

*S. lacrymans*	GO:0005975	BP	Carbohydrate metabolic process	5,8	1,1	0,000289	0,088
	GO:0006725	BP	Cellular aromatic compound metabolic process	2,5	0,2	0,00272	0,41
	GO:0016998	BP	Cell wall macromolecule catabolic process	1,7	0,1	0,00374	0,47
	GO:0008152	BP	Metabolic process	11,7	5,3	0,0059	0,554
	GO:0004553	MF	Hydrolase activity, hydrolyzing O-glycosyl compounds	5,8	0,6	6, 87*E* − 06	0,01
	GO:0016491	MF	Oxidoreductase activity	10,0	3,1	0,000372	0,092
	GO:0019131	MF	Obsolete tripeptidyl-peptidase I activity	1,7	0,1	0,00236	0,378
	GO:0003824	MF	Catalytic activity	11,7	5,3	0,00561	0,548
	GO:0018658	MF	Salicylate 1-monooxygenase activity	1,7	0,1	0,00846	0,656
	GO:0048037	MF	Cofactor binding	2,5	0,4	0,00858	0,688

^∗^Ratios correspond to percentage of genes with this associated GO term.

## Data Availability

The data used to support the findings of this study are included within the article.

## Supplementary Materials

Supplementary 1 Dataset S1: annotation of lncRNAs in *C. puteana*.

Supplementary 2 Dataset S2: annotation of lncRNAs in *S. lacrymans*.

Supplementary 3 Figure S3: IGV snapshot of *S. lacrymans* scaffold 3 (3.26 Mb), including reference annotation (blue), REPET-TE annotation (green), and lncRNAs (red).

Supplementary 4 Figure S4: distribution of transcriptional levels of coding transcripts and lncRNAs of *C. puteana* (Conpu) and *S. lacrymans* (Serla) cultured on MNN medium. Box plots inside the violin plots are presented, showing the median value as a horizontal line.

Supplementary 5 Figure S5: expression profiles of *S. lacrymans* DEG genes and their linked upstream lncRNAs showing opposite (A) and same (B) expression trends in SOM vs. minimal MNN media.

Supplementary 6 Table S6: annotation and expression ratios of gene-lncRNA pairs in *C. puteana* and *S. lacrymans.*
