# Supplementary figures and images for: Distribution, Characteristics, and Regulatory Potential of Long Noncoding RNAs in Brown-Rot Fungi
Source: Int J Genomics. 2019 May 2;2019:9702342. doi: 10.1155/2019/9702342 (PMC6525899; doi:10.1155/2019/9702342)

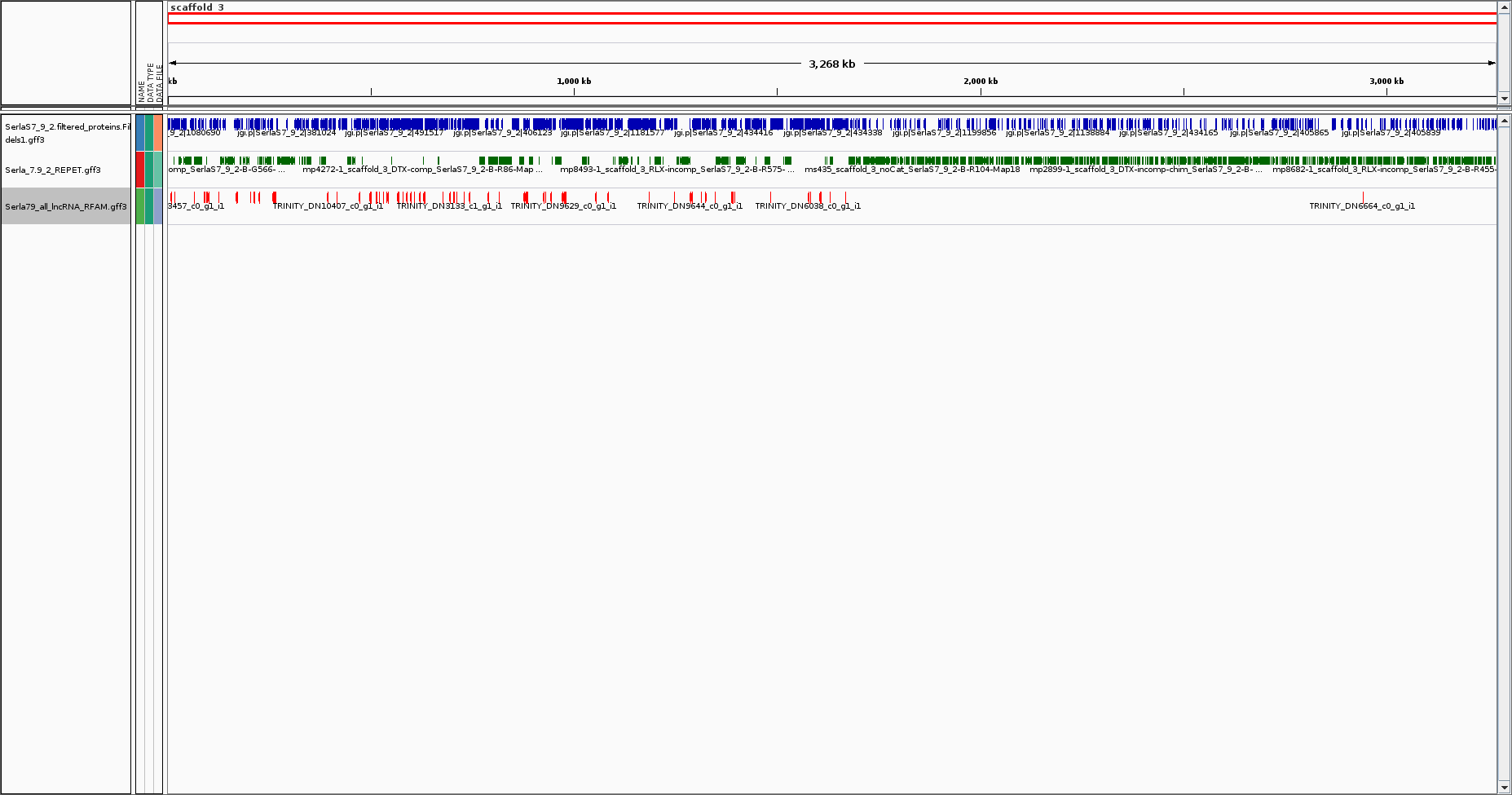

Supplement: Supplementary 3 — Figure S3: IGV snapshot of S. lacrymans scaffold 3 (3.26 Mb), including reference annotation (blue), REPET-TE annotation (green), and lncRNAs (red). [file 9702342.f3.png]

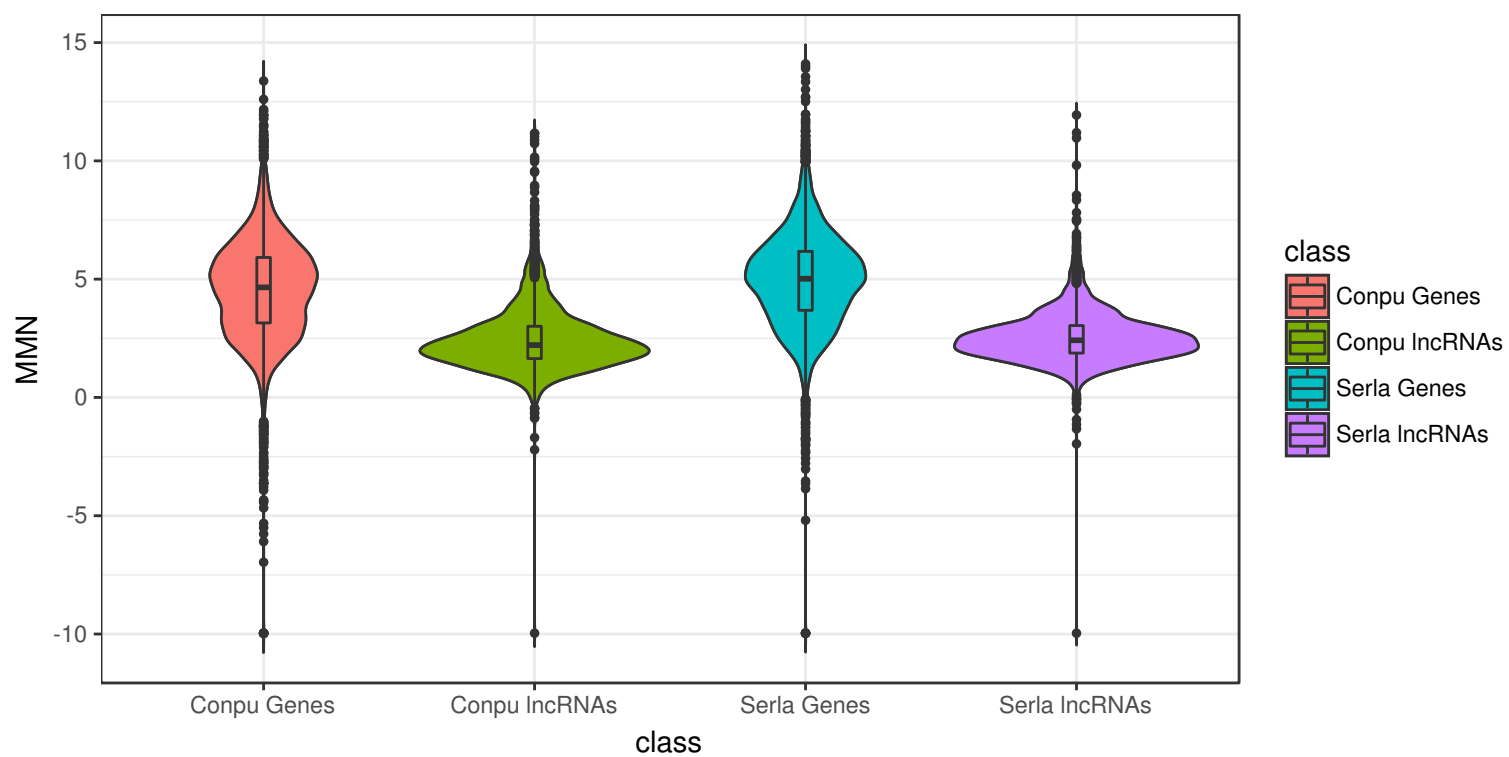

Supplement: Supplementary 4 — Figure S4: distribution of transcriptional levels of coding transcripts and lncRNAs of C. puteana (Conpu) and S. lacrymans (Serla) cultured on MNN medium. Box plots inside the violin plots are presented, showing the median value as a horizontal line. [file 9702342.f4.pdf]

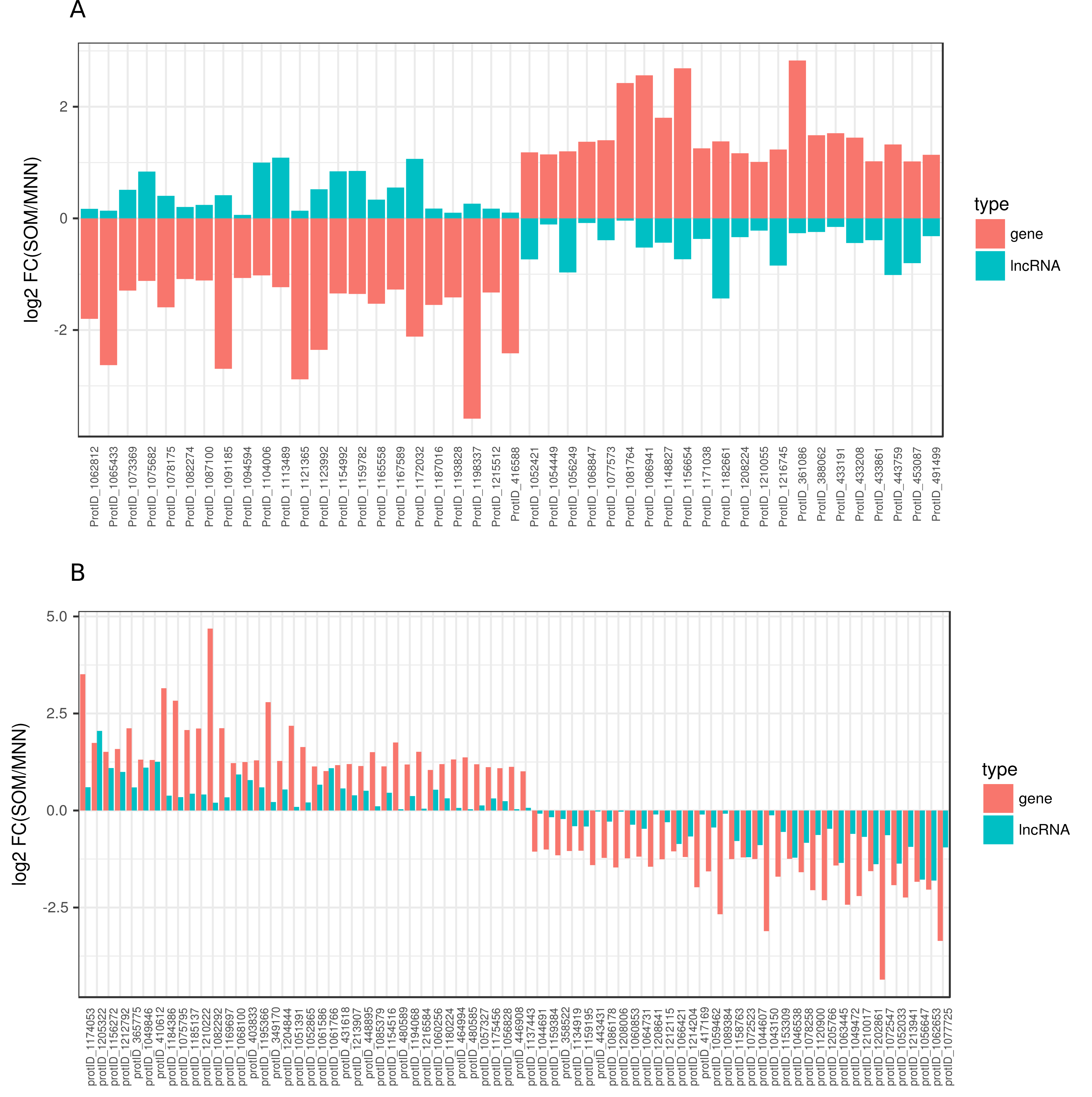

Supplement: Supplementary 5 — Figure S5: expression profiles of S. lacrymans DEG genes and their linked upstream lncRNAs showing opposite (A) and same (B) expression trends in SOM vs. minimal MNN media. [file 9702342.f5.png]
